# Genetic Variations in the *TP53* Pathway in Native Americans Strongly Suggest Adaptation to the High Altitudes of the Andes

**DOI:** 10.1371/journal.pone.0137823

**Published:** 2015-09-18

**Authors:** Vanessa Cristina Jacovas, Diego Luiz Rovaris, Orlando Peréz, Soledad de Azevedo, Gabriel Souza Macedo, José Raul Sandoval, Alberto Salazar-Granara, Mercedes Villena, Jean-Michel Dugoujon, Rafael Bisso-Machado, Maria Luiza Petzl-Erler, Francisco Mauro Salzano, Patricia Ashton-Prolla, Virginia Ramallo, Maria Cátira Bortolini

**Affiliations:** 1 Departamento de Genética, Instituto de Biociências, Universidade Federal do Rio Grande do Sul, Porto Alegre, Rio Grande do Sul, Brasil; 2 Consejo Nacional de Investigaciones Científicas y Tecnológicas (CONICET), Puerto Madryn, Argentina; 3 Facultad de Medicina Humana, Universidad de San Martin de Porres (USMP), Lima, Peru; 4 Instituto Boliviano de Biología de Altura (IBBA), Universidad Mayor de San Andres, La Paz, Bolivia; 5 Anthropologie Moléculaire et Imagerie de Synthèse, CNRS UMR 5288, Université Paul Sabatier Toulouse III, Toulouse, 31000, France; 6 Laboratório de Genética Molecular, Departamento de Genética, Universidade Federal do Paraná, Curitiba, PR, Brasil; 7 Serviço de Genética Medica, Hospital de Clínicas de Porto Alegre, Porto Alegre, RS, Brazil; University of Saarland Medical School, GERMANY

## Abstract

The diversity of the five single nucleotide polymorphisms located in genes of the *TP53* pathway (*TP53*, rs1042522; *MDM2*, rs2279744; *MDM4*, rs1563828; *USP7*, rs1529916; and *LIF*, rs929271) were studied in a total of 282 individuals belonging to Quechua, Aymara, Chivay, Cabanaconde, Yanke, Taquile, Amantani, Anapia, Uros, Guarani Ñandeva, and Guarani Kaiowá populations, characterized as Native American or as having a high level (> 90%) of Native American ancestry. In addition, published data pertaining to 100 persons from five other Native American populations (Surui, Karitiana, Maya, Pima, and Piapoco) were analyzed. The populations were classified as living in high altitude (≥ 2,500 m) or in lowlands (< 2,500 m). Our analyses revealed that alleles *USP7-*G, *LIF-*T, and *MDM2-*T showed significant evidence that they were selected for in relation to harsh environmental variables related to high altitudes. Our results show for the first time that alleles of classical *TP53* network genes have been evolutionary co-opted for the successful human colonization of the Andes.

## Introduction

The product of the *TP53* gene is a transcription factor (p53) that activates or represses a large number of target genes that regulate a broad array of extremely important cellular functions, such as cell cycle, metabolism, DNA repair, senescence, and apoptosis. This factor is therefore essential for maintaining genome integrity [1]. In humans, p53 has 393 amino acids and the *TP53* gene is located in the short arm of chromosome 17 [2]. Alterations of the *TP53* gene or perturbations in the *TP53* pathway are frequently correlated with carcinogenesis; more than 50% of human tumors carry mutations in this gene [3].

The steady-state levels of p53 are primarily determined by the rate at which it is degraded, rather than the rate at which it is produced. The *TP53* gene is constitutively expressed in all cell types, but p53 does not accumulate in non-stressed cells, since it is rapidly degraded by the proteasome via ubiquitination [4, 5]. On the other hand, the p53 levels increase in response to various stress signals, such as UV irradiation, low oxygen concentrations (hypoxia), and exposure to high temperatures [6, 7, 8, 9].

There are many polymorphisms described for *TP53*, but a C→G non-synonymous substitution (rs1042522: c.215C>G, p. Pro72Arg; [10]) that promotes the amino acid change Pro→Arg at codon 72 of p53 is one of the most widely studied. This polymorphism has been described to be associated with an increased risk for developing cancer, since the p53-Pro allele is less active than p53-72Arg in inducing apoptosis, among other characteristics [11, 12].

Proper p53 transcriptional function is strongly linked to the activity of several other proteins encoded by the genes *MDM2* (Mouse double minute 2 homolog; OMIM 164785), *MDM4* (Mouse double minute 4 homolog, OMIM 602704), and *USP7* (Ubiquitin-specific protease 7; OMIM 602519), also known as *HAUSP* (Herpesvirus-associated ubiquitin-specific protease). Another important gene in the so-called classical *TP53* network [13] is *LIF* (Leukemia-inhibitory factor; OMIM 159540), which plays an essential role in the early phases of embryonic development in humans, and is regulated by p53 (S1 Fig).

The E3 ubiquitin-protein ligase, MDM2, mediates the activity of p53 by directing it to degradation by the proteasome [5, 14, 15]. *MDM2* expression is also tightly regulated by p53 [16]. This auto-regulatory loop allows for the precise regulation of protein levels and activities of both p53 and MDM2 proteins [4, 17, 18].

The most well studied polymorphism in the *MDM2* gene (rs2279744: c.14+309T>G) is located in its internal promoter. It consists of a single-nucleotide change from T→G, which increases the affinity of a sequence in *MDM2* for the Sp1 transcription factor (Specificity protein 1; OMIM 189906). As a result, homozygotes for the G allele express more MDM2 than homozygotes for the T allele [19, 20]. In the presence of high levels of MDM2, there is a corresponding decrease of p53, causing a reduced response to cellular stress, impaired DNA repair, decreased apoptosis, and senescence [19]. Some studies have demonstrated that *MDM2-309T* and *MDM2-309G* alleles have different distributions in human populations [18, 21]. For instance, derived allele *MDM2-309-G* has higher frequency in European and Asian than African populations (average values: ∼0.35, ∼0.70, and ∼0.03, respectively; [22, 23, 24]). This allele may compensate for the higher apoptotic frequencies caused by the prevalence of allele p53-72Arg in Eurasians (∼0.56; [22, 23, 24]), suggesting adaptation [18].

The MDM4 protein, encoded by the *MDM4* gene acts as a negative regulator of p53, inhibiting its transcriptional activity [25, 26, 27]. MDM2 and MDM4 form heterodimers with a high capacity for ubiquitination of target proteins, thus leading to degradation of targets, like p53 [28]. Deletion of either *MDM2* or *MDM4* induces p53-dependent early embryonic lethality in an animal model [16, 29]. The AA genotype for the single-nucleotide *MDM4* polymorphism (rs1563828:g.204547449A>G) was associated with an increased risk for breast cancer [30].

Another important regulator of p53 is USP7, encoded by the *USP7* gene, which deubiquitylates p53 and protects it from proteasome degradation [31]. The *USP7* gene has a G→A substitution in intron 25 (rs1529916: g.8897333G>A), and derived allele A has been associated with endometriosis, female infertility, and prostate cancer [13, 32].

LIF is a cytokine expressed in various cell types, and its main function is to strengthen the blastocyst training of human embryos. In the very first days post-fertilization, LIF expression increases in the endometrium, creating a favorable environment for blastocyst implantation. Allele *G* of *LIF* (T→G transversion at the 3′ UTR region of the gene; rs929271: g.30242237T>G) is associated with female infertility [13]. LIF expression level is also known to be 2 times lower in cells bearing the p53-72Pro allele, compared to p53-72Arg, which can lead to the decrease of the implantation and fertility rate. In summary, several studies have strongly suggested that polymorphisms in the p53 signaling pathway play an important role in blastocyst implantation and are associated with recurrent pregnancy loss [13, 33, 34].

The genetic variability observed in contemporary human populations and the functionalities associated with the polymorphisms described above allow us to infer that a simple neutral model of mutation and drift is insufficient to explain the allelic distributions observed. Thus, it has been suggested that positive selection contributed to adaptation of *Homo sapiens* in different ecosystems. For example, the p53-72Arg allele (rs1042522) is more common in Europeans than in Africans, leading to the hypothesis that its distribution is dependent on latitude and maintained by selective pressures [35, 36]. On the other hand, Shi *et al*. [23] found that winter temperatures and UV radiation correlated significantly with the *TP53* (rs1042522) and *MDM2* (rs2279744) allele distributions in East Asian populations, indicating the possibility of adaptation to distinct environments.

America was the last continent occupied by humans in pre-colonial times. González-José *et al*. [37] and Bortolini *et al*. [38] suggested that an initial major dispersal began after 21,000 years before present, and that the biological and cultural characteristics of the first Americans that emerged, in part, were reshaped by recurrent trans-Beringian/circum-Arctic gene flow and important local population dynamics during a standstill period in Beringia. For example, Native Americans have experienced dramatic episodes of genetic drift and successive bottleneck events during migration across the continent. Furthermore, signals of positive natural selection associated to autochthonous environmental and cultural conditions have also been described [39, 40, 41].

Based on these findings, we hypothesize that the allele distributions of the classical *TP53* pathway genes in Native American populations reflect adaptation, not only demographic and/or random events. To test our hypothesis, we determined the genotypes of the five above-mentioned SNPs in 282 unrelated individuals and compared the results to a large number of climate-related environmental variables, such as altitude, temperature, and seasonal mean UV radiation. Additional data regarding two of these SNPs (*TP53-*rs1042522 and *MDM2-*rs2279744) were compiled from the literature for a more extensive population analysis.

## Materials and Methods

### Samples and ethical procedures

Five SNPs (rs929271, rs1042522, rs1563828, rs2279744, and rs1529916) were genotyped in 282 volunteers characterized as Native American or as having large (> 90%; [42]) Native American ancestry. Volunteers were from 12 populations located in different ecoregions, namely highland (populations located at altitudes ≥ 2,500 m; [43]) and lowland (populations located at altitudes below 2,500 m). Highland populations were Aymara (n = 18) and Quechua (n = 17) from Bolivia, and Chivay (n = 18), Cabanaconde (n = 17), Yanke (n = 10), Taquile (n = 43), Amantani (n = 29), Anapia (n = 15), and Uros (n = 22) from Peru. All highland populations were located in the Andean region, including on Lake Titicaca islands or in their vicinity. Lowland populations were Andoas (n = 61), a Native Amazonian population living in North Peru, and Guaraní Indians from Brazil (Tupian speakers from two sub-groups: Ñandeva, n = 16; and Kaiowa, n = 16). Details about these populations have been summarized elsewhere [42, 44, 45, 46]. To facilitate the presentation of the results and discussion, we will collectively refer to all communities as “Native Americans”. The geographical coordinates (latitude and longitude) of all populations are presented in S1 File (Table A in S1 File).

Ethical approval for the use of these samples was obtained from the National Ethics Committee of Brazil (Resolution No. 123/98 CONEP) for individuals from Brazilian tribes; and by the Ethics Committee of Universidad San Martín de Porres, Lima, Peru (Peruvian samples) and Université Paul Sabatier Toulouse, Toulouse, France (Bolivian samples). Written informed consent or verbal informed consent (illiterate persons) was obtained individually from tribal participants. Verbal informed consent was registered in the field, and the institutional review ethics committees approved this procedure. This study was carried out in accordance with the Declaration of Helsinki.

### Data from literature

Data from 100 additional individuals from five other Amerindian populations (Surui and Karitiana (Brazil), Piapoco (Colombia), Maya and Pima (México) were included in this study. For more details on these samples, please refer to http://www.cephb.fr/HGDP-CEPH-Panel/[47]. The environmental conditions evaluated for all populations (present study and literature sample) are compiled in S1 File (Table A in S1 File). The environmental data were collected for each population using the SoDa Service and WorldClim (http://www.soda-is.com/[48] and http://www.worldclim.org/[49], respectively; last access: December 19, 2014).

All analyses were performed with two sets of data: (A) 12 South American populations, for which original data regarding five SNPs (rs1042522, rs2279744, rs1529916, rs1563828, and rs929271) were obtained in the present study, and (B) all populations genotyped in this study plus five additional populations, for which *TP53* rs1042522 and *MDM2* rs2279744 data are available in the HGDP-CEPH panel [22].

### Laboratory methods

Genomic DNA was obtained from saliva, whole blood, or plasma, using the QIAamp DNA extraction Mini kit (Qiagen; https://www.qiagen.com/br/[50]) according to manufacturer’s instructions. Genotyping of the *TP53-*rs1042522, *MDM4-*rs1563828, *USP7-*rs1529916, *LIF-*rs929271, and *MDM2-*rs2279744 SNPs was performed by allelic discrimination using the TaqMan Genotyping Assays (Applied Biosystems; http://www.lifetechnologies.com/br/en/home/brands/applied-biosystems.html [51]). Genotyping of *MDM2* rs2279744 was performed using a customized (assay-by-design) assay using probes FAM-TCCCGCGCCGCAG and VIC-CTCCCGCGCCGAAG, with primers 5′-CGGGAGTTCAGGGTAAAGGT-3′ (forward) and 5′-ACAGGCACCTGCGATCATC-3′ (reverse).

PCR reactions were carried out in 48-well plates, with each reaction containing: 10 ng of genomic DNA, 2× TaqMan® genotyping Master Mix (Applied Biosystems), specific probes for each SNP (40×), and ultra-pure water for a final reaction volume of 10 μL. The PCR conditions were as follows: 95°C for 10 min, followed by 45 cycles of 95°C for 15 s and 63°C for 60 s. *MDM2* rs2279744 genotyping was also done in 48-well plates, with each reaction containing: 10 ng of genomic DNA, 2× TaqMan® genotyping Master Mix, 5 μM of each primer and probe, and water to reach a final volume of 10 μL. *MDM2* PCR conditions were as follows: 50°C for 2 min, 95°C for 10 min, and 45 cycles of 95°C for 15 s and 60°C for 60 s. All reactions were performed in an Illumina Eco Real-Time PCR System, (http://www.uniscience.com/ [52]) and results were analyzed using an Eco Real-Time PCR System and the Software v5.0 associated with that system. All wet-lab analyses were performed in the Laboratory of Human and Molecular Evolution of the Department of Genetics at Federal University of Rio Grande do Sul in Brazil.

### Statistical analyses

Hardy-Weinberg equilibriums were calculated using a web-based program (http://www.oege.org/software/hwe-mr-calc.shtml [53]), and the statistical significance was assessed by Chi-square tests (*p* < 0.01). Analysis of molecular variance (AMOVA) using Arlequin 3.5.1.227 was applied to assess the variance among and within the investigated Native American populations [54, 55, 56].

Allele distributions were tested for possible associations with three groups of environmental conditions: 1) geographic: altitude, latitude, and longitude; 2) annual and seasonal mean UV radiation, and 3) Nineteen climate-related variables (Table A in S1 File). Principal component analysis (PCA) was performed to convert the nineteen possibly correlated bioclimatic variables into a smaller number of artificial variables (PCs) accounting for most of the variance in the previously observed variables. The correlation analysis between allele frequencies in each population and the environmental conditions was performed using Spearman´s rho correlation coefficient. The association between SNPs and altitude was assessed through binary logistic regression using two geographic categories (highlands: ≥ 2,500 m, and lowlands: < 2,500 m [43]) as the outcome and SNPs as predictors. Since this analysis was not intended to infer causality relationships, the odds ratio was reported as an estimate of size effect. For these analyses a Bonferroni correction was performed and the alpha was set at 0.01 (α_Bonferroni_ = 0.05/5 SNPs tested). Additionally, we performed the nonparametric Multifactor Dimensionality Reduction (MDR, v3.0.2; [57]) approach to detect potential gene–gene interactions. Thus, we used MDR to incorporate information from our 5 and 2 selected loci (data sets A and B, respectively) and an environmental condition as the outcome (altitude: highland and lowland geographic categories). The percentage of information gain (IG) by each SNP is visualized for each node, while the IG for each pairwise connection between SNPs is visualized for each branch. Thus, the independent main effects of each SNP can be compared to the interaction effect. The *p*-value was calculated based on 10,000 permutations.

## Results

Table 1 shows the derived allele frequency for each SNP investigated (individual genotypes can be seen in S2 File). Wide variations were observed in some allele frequencies in both population groups (highland and lowland). For instance, the frequency of MDM2-309-G is about five times higher in Guaraní Ñandeva than Guaraní Kaiowa, which may reflect genetic drift since the split of these two Guaraní partialities occurred less than 2,000 years ago [45]. On the other hand, several highland populations from Peru and Bolivia present similar distributions of *MDM2-309-G*. Most of these highland populations show deviations from the Hardy-Weinberg equilibrium (HWE), especially in Peruvian samples for the *MDM2*-309 locus (Table B in S1 File).

**Table 1:** Derived^1^ allele frequencies and AMOVA results.

Population (n)	*TP53* G rs1042522	*MDM2* G rs2279744	*MDM4* G rs1563828	*USP7* A rs1529916	*LIF* G rs929271	Reference
Highlands (≥2.500 m.)						
Amantani (29)	0.86	**0.13**	0.60	0.39	0.77	This study
Anapia (15)	0.77	**0.13**	0.57	0.30	0.47	This study
Cabanaconde (17)	0.88	**0.12**	0.74	0.09	0.32	This study
Chivay (18)	0.83	**0.14**	0.67	0	0.36	This study
Taquile (43)	0.92	**0.05**	0.63	0.23	0.44	This study
Uros (22)	0.93	**0.20**	0.84	0.05	0.27	This study
Yanke (10)	0.65	0.05	0.80	0.10	0.45	This study
Aymara (16–18)^2^	0.78	0.21	0.50	0.14	0.12	This study
Quechua (15–17)^2^	**0.53**	0.21	0.66	0.24	**0.40**	This study
***Fst***	0.068, *p* = 0.017	-0.020, *p* = 0.801	-0.006, *p* = 0.506	0.068, *p* = 0.013	0.118, *p*<0.001	
Lowlands (<2.500 m.)						
Andoas (61)	0.74	**0.16**	0.53	0.39	0.59	This study
Guarani Kaiowa (16)	0.94	0.07	0.66	0.34	0.56	This study
Guarani Ñandeva (15–16)^2^	0.87	0.33	0.72	0.57	0.63	This study
Karitiana (23–24)^2^	0.62	0.59	ND	ND	ND	Sucheston *et al*. 2011
Maya (23–21)^2^	0.83	0.64	ND	ND	ND	Sucheston *et al*. 2011
Piapoco/Curripaco (12–13)^2^	0.92	0.81	ND	ND	ND	Sucheston *et al*. 2011
Pima (21–24)^2^	0.65	0.69	ND	ND	ND	Sucheston *et al*. 2011
Surui (17–20)^2^	1	**0.32**	ND	ND	ND	Sucheston *et al*. 2011
***Fst***	0.054, *p* = 0.028	0.274, *p*<0.001	0.029, *p* = 0.200	0.020, *p* = 0.243	-0.044, *p* = 1.000	
***Fct***	-0.008, *p* = 0.516	0.111, *p* = 0.029	0.001, *p* = 0.413	0.091, *p* = 0.043	0.020, *p* = 0.261	

^1^Defined in comparison with the Chimpanzee sequence. For the loci in **bold** deviations from Hardy-Weinberg Equilibrium were detected.

^2^The number of individuals vary according to the investigated locus. ND: No data available.

AMOVA analysis, using both data sets (Table 1), indicated that homogeneity and population structures could be seen in both highland and lowland populations. For instance, population structure measured by F_ST_ statistics (*i*.*e*. the among-populations component of genetic variance) is observed in the two groups considering *TP53* rs1042522 (F_ST_ = 0.068 and 0.054, for highland and lowland, respectively), while for *MDM2* rs2279744 homogeneity is observed in highland populations (F_ST_ = −0.020; *p* = 0.801) while high heterogeneity is observed in lowland populations (F_ST_ = 0.274; *p* < 0.001). Only the F_ST_ value observed for *LIF* rs929271 in the highland group (11.8%) is similar to the average estimated across the human genome (12%; [58]). For F_CT_ (between-groups component of variance), the variance is high (11%) for *MDM2* rs2279744 data, indicating a remarkable and significant difference between the allelic distributions of the highland and lowland populations.

### Principal component analysis

In data set A, the first principal component (PC1) accounted for 73% of total variance, comprising the following bioclimatic variables: annual mean temperature, mean diurnal range, maximum temperature of warmest month, minimum temperature of coldest month, temperature annual range, mean temperature of wettest quarter, mean temperature of driest quarter, mean temperature of warmest quarter, mean temperature of coldest quarter, annual precipitation, precipitation of wettest month, precipitation in the driest month, precipitation seasonality, precipitation of wettest quarter, precipitation of driest quarter, precipitation of warmest quarter, and precipitation of coldest quarter. The second principal component (PC2) represented 13% of variance, and comprised temperature seasonality, which is a measure of standard deviation × 100 of average annual daily temperatures.

When we expanded our analysis to data set B, PC1 represented 59% of total variance and comprised sixteen bioclimatic variables: annual mean temperature, mean diurnal range, maximum temperature of warmest month, minimum temperature of coldest month, mean temperature of wettest quarter, mean temperature of driest quarter, mean temperature of warmest quarter, mean temperature of coldest quarter, annual precipitation, precipitation of wettest month, precipitation in the driest month, precipitation seasonality, precipitation of wettest quarter, precipitation of driest quarter, precipitation of warmest quarter, and precipitation of coldest quarter. The second principal component (PC2) represented 23% of variance, and comprised isothermality (the ratio of mean diurnal range to temperature annual range), temperature seasonality, and temperature annual range, all of which are connected with climatic changes by seasonality.

### Correlation analyses

Correlation coefficients and their statistical significances are given in Table 2. In data set A, there were significant associations between the G allele of *USP7* (rs1529916) and the annual mean of ultraviolet irradiance (rho = 0.760 *p* = 0.004) and PC1 (rho = −0.741, *p* = 0.006). This allele was also nominally associated with the mean of ultraviolet irradiance in the coldest semester (rho = 0.681 *p* = 0.015) and in the warmest semester (rho = 0.618 *p* = 0.032). *MDM2* (rs2279744) T allele was nominally associated to longitude (rho = −0.587 *p* = 0.045) and the mean of ultraviolet irradiance in the coldest semester (rho = 0.605 *p* = 0.037), while *LIF* (rs929271) T allele was nominally associated to annual mean of ultraviolet irradiance (rho = 0.693 *p* = 0.013) and PC1 (rho = −0.664, *p* = 0.018).

**Table 2:** Correlation analysis results.

Variables	*TP53* C		*MDM2* T		*MDM4* A		*USP7* G		*LIF* T	
	rs1042522		rs2279744		rs1563828		zrs1529916		rs929271	
	rho	*p*-value	rho	*p*-value	rho	*p*-value	rho	*p*-value	rho	*p*-value
**Data set A**										
Altitude	0.091	0.778	0.442	0.150	0.330	0.295	0.291	0.359	0.312	0.324
Latitude	0.503	0.095	0.380	0.224	-0.074	0.820	0.301	0.342	0.140	0.665
Longitude	-0.161	0.618	-0.587	*0*.*045*	0.322	0.307	-0.371	0.236	-0.035	0.914
UV irradiance 1	0.092	0.776	0.542	0.069	-0.143	0.657	0.760	**0.004**	0.693	*0*.*013*
UV irradiance 2	0.190	0.553	0.605	*0*.*037*	-0.120	0.710	0.681	*0*.*015*	0.497	0.100
UV irradiance 3	-0.042	0.897	0.369	0.238	-0.165	0.608	0.618	*0*.*032*	0.516	0.086
PC1	-0.140	0.665	-0.390	0.210	0.109	0.737	-0.741	**0.006**	-0.664	*0*.*018*
PC2	-0.140	0.665	-0.464	0.129	-0.175	0.586	-0.049	0.880	0.028	0.931
**Data set B**										
Altitude	0.006	0.981	0.673	**0.003**	-	-	-	-	-	-
Latitude	0.277	0.281	-0.292	0.255	-	-	-	-	-	-
Longitude	-0.293	0.253	-0.270	0.294	-	-	-	-	-	-
UV irradiance 1	0.265	0.304	0.410	0.102	-	-	-	-	-	-
UV irradiance 2	0.147	0.574	0.827	**<0.001**	-	-	-	-	-	-
UV irradiance 3	0.215	0.408	0.245	0.343	-	-	-	-	-	-
PC1	-0.177	0.497	-0.610	**0.009**	-	-	-	-	-	-
PC2	-0.015	0.955	-0.567	*0*.*018*	-	-	-	-	-	-

Nominal associations are depicted in *italics* and significant associations (after Bonferroni correction) are depicted in **bold**.

UV irradiance 1: annual mean of ultraviolet irradiance, UV irradiance 2: mean of ultraviolet irradiance in the coldest semester, UV irradiance 3: mean of ultraviolet irradiance in the warmest semester.

PC = Principal Component.

In data set B, there were significant associations between the T allele of *MDM2* (rs2279744) and altitude (rho = 0.673, *p* = 0.003), the mean of ultraviolet irradiance in the coldest semester (rho = 0.827 *p* < 0.001), and PC1 (rho = −0.610 *p* = 0.009). This allele was also nominally associated to PC2 (rho = −0.567, *p* = 0.018).

### Binary logistic regression analyses

We performed a binary logistic regression analysis to search for possible associations between SNPs and two geographic categories (Highlands: ≥ 2,500 m; Lowlands: < 2,500 m) using altitude as dependent variable (Table C in S1 File). In data set A, we observed statistically significant associations for *USP7* rs1529916 and *LIF* rs929271 SNPs. Individuals who inhabit the highlands were less likely to carry *USP7*-GA (OR = 0.417, *p* = 0.002) and *USP7*-AA (OR = 0.135, *p* < 0.001) genotypes. A similar association was observed for *LIF*-TG (OR = 0.324, *p* = 0.001 and *LIF*-GG (OR = 0.270, *p* < 0.001) genotypes. Regarding data set B, an association between the *MDM2* rs2279744 SNP and altitude was detected. Individuals who inhabit the highlands were less likely to carry *MDM2*-TG (OR = 0.218, *p* < 0.001) and *MDM2*-GG (OR = 0.175, *p* < 0.001) genotypes.

### Gene-gene interaction analyses

**Fig 1:**
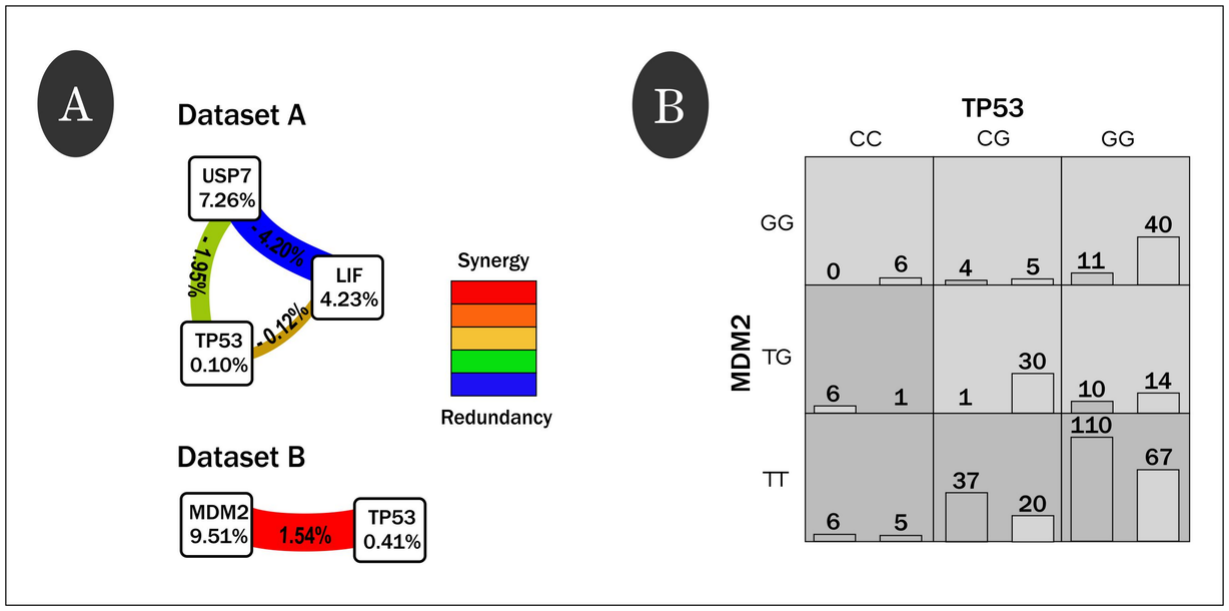
Summary of the multifactor dimensionality reduction (MDR) interaction models. **(A)** Interaction graphs comprised of nodes with pairwise connections between them. Values in nodes represent information gain (IG) of individual genes (main effect), while values between nodes are the IG of each pairwise combination (interaction effects). Positive entropy (plotted in red) indicates interaction (epistasis) and negative entropy (plotted in green or blue) indicates redundancy. Independence is represented by the gold color. **(B)** The *MDM2*-*TP53* interaction associated with altitude in data set B. High-frequency genotype combinations in individuals who inhabit highlands (≥ 2,500 meters) are depicted as darkly shaded cells and low-frequency combinations in those individuals as lightly shaded. For each cell, the left bar indicates the absolute number of individuals who inhabit highlands and the right bar the absolute number of individuals who inhabit lowlands (< 2,500 meters).

We used the MDR approach to search for gene-gene interactions (Table D in S1 File). These analyses were intended to explore differences between highland and lowland populations in genotype combinations among the SNPs investigated since gene networks, such as those investigated here, can be sources of epistasis. Significant two- (*p* = 0.004) and three-locus interactions (*p* = 0.004) were identified in data set A. However, an analysis of IG based on entropy measures revealed that these effects were not explicated by epistasis (negative values in the branches among nodes; Fig 1A). On the other hand, IG values of both *USP7* (7.26%) and *LIF* (4.23%) indicated that both genes have a large main effect in a scenario where altitude is considered, corroborating our previous analysis. Regarding data set B, the largest main effect was observed for *MDM2* (IG = 9.51%), which contrasts with the low value for *TP53* (IG = 0.41%). A potential synergism (epistasis) between the two loci was also found, but it is apparently weak (IG value of only 1.54%; Fig 1A), at least when it is compared with the potential mechanism of action on *MDM2*. On the other hand, it is 3.7 times greater than the main effect of *TP53*. It is noteworthy that independent of the *TP53* genotype, the genotype *MDM2*-TT is always favorable and most commonly found in highlands (Fig 1B). In other words, *MDM2* showed the greatest contribution to adaptation to hostile environments, such as those found in the highlands.

## Discussion

More than 60,000 scientific studies have been published in the last 30 years concerning the roles of *TP53* network genes, as well as of their variants, in human susceptibility to cancer and other pathological conditions. Special issues in scientific journals, dedicated to these topics, have also been published (see, as example [59]). This overwhelming number of studies contrasts with the rarity of studies of an evolutionary context, which are indispensable for explaining differences in the *TP53* network allele distributions along human populations, which often cannot be understood as simply a result of stochastic processes. Our goal here was to help fill this gap, providing information about five polymorphisms of the classical *TP53* network in Native American populations and how their variability patterns could be explained.

Our analysis of data set A, which included original information of 5 SNPs in 12 Native American populations, suggests a well-known role of genetic drift in those groups, illustrated by wide difference in *MDM2*-G allele frequencies between the two Guaraní sub-groups. However, other instigating results can be associated to adaptation to environmental conditions in Native American populations. Alleles *USP7*-G (rs1529916) and *LIF*-T (rs929271) were correlated with ultraviolet irradiance and index of temperature and precipitation, variables comprising PC1. Additionally, examining variables with the highest representation in the PC1 components (> 0.90), it is possible to see that in regions where the annual mean temperatures, minimum temperatures of the coldest month, mean temperatures of the driest quarter, mean temperatures of the coldest quarter, and precipitation are low, the presence of ancestral alleles G and T are significantly higher. In other words, our analysis as whole reveals that alleles *USP7*-G and *LIF*-T are more highly represented in stressful environments (low temperature, arid climate, wide temperature range during the day, and high levels of UV radiation), which is typical of high altitudes. It is noteworthy that derived alleles of these SNPs have been associated with cancer susceptibility, infertility, and endometriosis [13, 32], so that the alleles *USP7*-G and *LIF*-T could be considered as protective factors against the consequences of harsh environmental stress.

Human populations living at high altitudes are likely to have developed specific adaptations to support both the harsh conditions described above and low oxygen concentrations (hypoxia; [41]). Monge in 1948 [60] proposed that the hypoxia could reduce fertility in humans. However, recent studies have shown that the reproductive functioning of populations indigenous to high altitudes is adapted to hypoxia and other extreme conditions [61]. Our results with *USP7* (rs1529916) and *LIF* (rs929271) polymorphisms could be connected with adaptation of the reproductively successful ancestors of modern Andes populations.

In examining data set B, we found the ancestral *MDM2*-T allele is strongly correlated with winter mean UV radiation, altitude, and PC1. The highest representations in the PC1 components (> 0.90) are annual mean temperature, minimum temperature of coldest month, minimum temperature of coldest quarter, and annual precipitation. Allele T is significantly more frequent in communities located at high altitudes experiencing extreme environmental conditions, such as high UV radiation and dry and cold climate. In addition, the binary logistic regression analysis showed that *MDM2*-TT individuals are more frequently found in highlands. *MDM2-*TT homozygotes express typical steady-state levels of MDM2, maintaining an adequate level of p53 [20], and consequently can appropriately respond environmental stresses. An important confounding factor could be admixture with Europeans, which is more important in Andean than in the lowland populations considered here [42, 62, 63]. However, any effect of admixture would be in the opposite direction, since *MDM2-*G frequency is relatively high in Spaniards (0.39; [24]).

The inverse correlation between *MDM2*-T frequencies and winter UV radiation is consistent with the findings of Shi *et al*. [23], which showed that low levels of UV are significantly correlated with genotype *MDM2-*GG in Han Chinese populations, similarly deviating from HWE. These authors suggested that *MDM2*-GG is selected for in areas of low UV activity (at high altitudes, the thinner atmosphere will filter less UV radiation; consequently for every 1000 m increase in altitude, the UV radiation level will increase ∼12%; http://www.weather.gov.hk/radiation/tidbit/201012/uv_e.htm [64]). Natural selection can be evoked to explain these results, although the HWE test is considered too weak to detect this phenomenon.

As mentioned above, native Andean populations have successfully adapted to environments with low oxygen concentrations. One gene that contributes to hypoxia adaptation is *EPAS1* (Endothelial PAS domain-containing protein 1, also known as *HIF-2α*, Hypoxia-inducible factor—alpha 2 (OMIM 603349)), which acts by preventing toxicity promoted by hypoxia. This gene plays an important role in both the classical and the expanded *TP53* network. For instance, the alpha subunit of EPAS1 regulates p53 activity, including through prevention of damage-induced degradation and nuclear export of MDM2, stabilizing nuclear p53 [65]. Foll *et al*. [41] confirmed the action of positive selection on *EPAS1* in both Tibetans and Andeans. Furthermore, several studies have revealed a role for p53 and its regulation in physiological and metabolic processes resulting from environments with low oxygen concentrations [8, 66, 67]. Recently, Eichstaedt *et al*. [68] studied an indigenous population living in the Argentinean Andes (Colla) and identified signatures of positive selection in genes involved in cellular hypoxia, including *TP53*. Importantly, hypoxia induces p53 accumulation through down-regulation of MDM2 [66]. These results reinforce our suggestion that individuals with the *MDM2-*TT genotype represent an adaptation to the environmental stresses of high altitudes. In addition, the interaction analysis performed by the MDR method using both data sets (A and B) revealed the potential for the *MDM2*, *LIF*, and *USP7* genes to play an additional central role in a high altitude setting. Thus, taken together, our results demonstrate that variation of the p53-activating stressors could not be directly correlated with p53-Pro72Arg alleles, but with frequencies of the other functional polymorphisms examined, such as *USP7*-G (rs1529916), *LIF*-T (rs929271), and *MDM2*-309, as well as synergic interactions between them.

Under neutral model conditions, South Amerindians living in lowlands present higher levels of population structure when compared to those seen in indigenous Andean communities [62, 69]. However, not all F_ST_ values obtained in our study were consistent with this expectation (Table 1). Positive selection disturbs the patterns of genetic variation expected under a standard neutral model [70]. Additionally, it is possible to see that some derived alleles, such as *MDM2-*G, have high frequencies in Asian populations with putative common ancestry (0.57–0.82; [22, 23, 24]), but a surprisingly low distribution in Andeans (average value: ∼0.13). An excess of unexpectedly low and/or high frequencies of derived alleles can also be considered a marker of positive selection [70]. Thus, the distributions of the classical *TP53* pathway alleles in Native American populations could be under selective pressure. Sucheston *et al*. [22] investigated 52 worldwide populations from the HGDP-CEPH-panel for the prevalence of p53-Pro72Arg and *MDM2*-309 polymorphisms, but found no significant association with climate variables. However, the Native American samples in the Sucheston *et al*.’ study [22] were much smaller than the present study (see Table 1), which may explain the divergent results.

Finally, government surveys in Peru indicate that the rate of gestational and postpartum complications in Aymara regions is lower than the national average (1.8% and 5% respectively; http://www.dge.gob.pe/publicaciones/pub_asis/asis26.pdf, p. 165; [71] http://www.dge.gob.pe/portal/docs/intsan/asis2012.pdf, p. 76 [72]). These same official sources also indicate differences in the cancer incidences between lowland localities and some regions situated at high altitude (for example in the Puno state, where the Anapia community is located; http://www.dge.gob.pe/portal/docs/asis_cancer.pdf, p. 64 [73]). These findings are in agreement with our genetic results. However, only additional and specific studies can accurately relate our evolutionary findings with those related to the health of contemporary Andean populations.

A well-regulated p53 network is crucial for maintaining genomic integrity. Several polymorphisms in this pathway have been described, and the different allele frequencies among human populations have been interpreted as the result of selective pressure. Humans occupied high-altitude locations in the Andes as early as 12,800 years ago, providing a sufficient period of time for the initiation of organismal selection and developmental functional adaptation ([74] and references therein). Here we are suggesting that natives from Andes, who are subjected to low temperatures, arid climates, wide temperature ranges during the day, high levels of UV radiation, and hypoxia, among other environmental insults, are protected by a selected combination of alleles/genotypes of the *TP53* pathway. The present study identifies for the first time the potential role of the *MDM2*, *LIF*, and *USP7* in the adaptation of the Andean populations.

## Supporting Information

S1 Fig The p53 network. Network view of p53 pathway analyzed by STRING 10.0 (http://string-db.org/). Interaction confidence score cutoff was 900 (highest confidence). Each color arrow represents a predicted functional partner: green (activation), red (inhibition), blue (binding), purple (catalysis), pink (post-translational modification), black (reaction), and yellow (expression). TP53 = tumor protein p53, USP7 = ubiquitin specific peptidase 7 (herpes virus-associated), MDM4 = Mouse double minute 4 homolog, MDM2 = Mouse double minute 2 homolog, and LIF = leukemia inhibitory factor.
(TIFF)

S1 File Additional Results. Climatic variables evaluated in population of this study **(Table A)**. Allelic frequencies and Hardy-Weinberg Equilibrium results **(Table B)**. Binary logistic regression analyses results **(Table C)**. Locus interaction by the multifactor dimensionality reduction (MDR) approach **(Table D)**.
(DOC)

S2 File Individual Genotype Database. In this database, we found individual genotypes of all polymorphisms discussed in the study.
(XLSX)
