# Supplementary figures and images for: Genetic Variations in the TP53 Pathway in Native Americans Strongly Suggest Adaptation to the High Altitudes of the Andes
Source: PLoS One. 2015 Sep 18;10(9):e0137823. doi: 10.1371/journal.pone.0137823 (PMC4575214; doi:10.1371/journal.pone.0137823)

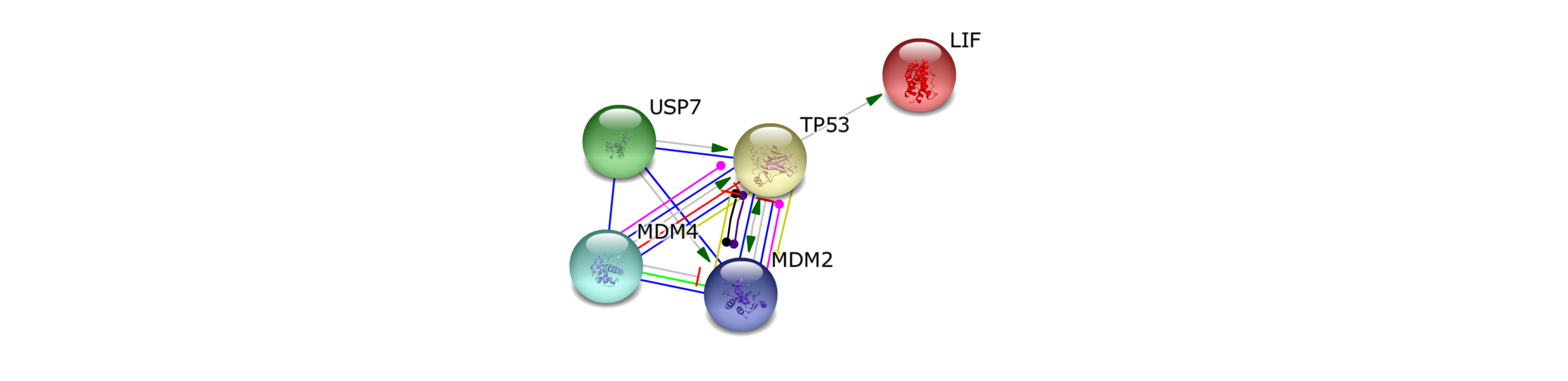

Supplement: S1 Fig — Network view of p53 pathway analyzed by STRING 10.0 (http://string-db.org/). Interaction confidence score cutoff was 900 (highest confidence). Each color arrow represents a predicted functional partner: green (activation), red (inhibition), blue (binding), purple (catalysis), pink (post-translational modification), black (reaction), and yellow (expression). TP53 = tumor protein p53, USP7 = ubiquitin specific peptidase 7 (herpes virus-associated), MDM4 = Mouse double minute 4 homolog, MDM2 = Mouse double minute 2 homolog, and LIF = leukemia inhibitory factor. (TIFF) [file pone.0137823.s001.tiff]
