# Supplementary material for: Genetic Variations in the TP53 Pathway in Native Americans Strongly Suggest Adaptation to the High Altitudes of the Andes
Source: PLoS One. 2015 Sep 18;10(9):e0137823. doi: 10.1371/journal.pone.0137823 (PMC4575214; doi:10.1371/journal.pone.0137823)
Supplement: S1 File — Climatic variables evaluated in population of this study (Table A). Allelic frequencies and Hardy-Weinberg Equilibrium results (Table B). Binary logistic regression analyses results (Table C). Locus interaction by the multifactor dimensionality reduction (MDR) approach (Table D). (DOC) [file pone.0137823.s002.doc]

**S1. Sprtnional Results.**

**S1 File. Additional Results.**

**Table A.** Climatic variables evaluated in population of this study.

| Populations | Aymara | Quechua | G. Ñandeva | G. Kaiowá | Amantani | Andoas | Anapia | Cabanaconde | Chivay | Taquile | Uros | Yanke | Karitiana | Maya | Pima | Surui | Piapoco/Curripaco |
| --- | --- | --- | --- | --- | --- | --- | --- | --- | --- | --- | --- | --- | --- | --- | --- | --- | --- |
| Altitude | 4312 | 2656 | 319 | 456 | 3827 | 235 | 4366 | 3441 | 3820 | 3825 | 3818 | 3820 | 86 | 3 | 361 | 303 | 117 |
| Latitude | -19.25 | -14.5 | -23.8 | -23.1 | -15.65 | -2.9 | -16.31 | -15.62 | -15.63 | -15.76 | -15.74 | -15.64 | -8.75 | 20.22 | 33.17 | -8.4 | 3.0 |
| Longitude | -69.08 | -69.0 | -54.5 | -55.2 | -69.71 | -76.4 | -68.85 | -71.98 | -71.98 | -69.68 | -69.93 | -71.95 | -63.84 | -90.47 | -11.87 | -54.9 | -68.0 |
| UV irradiance 1 | 230 | 202 | 212 | 210 | 212 | 206 | 212 | 248 | 224 | 216 | 214 | 226 | 180 | 216 | 246 | 180 | 162 |
| UV irradiance 2 | 210 | 198 | 150 | 154 | 206 | 209 | 204 | 241 | 214 | 206 | 206 | 214 | 194 | 184 | 183 | 176 | 172 |
| UV irradiance 3 | 252 | 205 | 212 | 210 | 220 | 203 | 219 | 219 | 235 | 220 | 221 | 238 | 165 | 246 | 309 | 161 | 153 |
| Bio 1 | 32 | 166 | 218 | 221 | 83 | 257 | 86 | 132 | 83 | 84 | 85 | 93 | 260 | 267 | 212 | 253 | 267 |
| Bio 2 | 181 | 113 | 117 | 118 | 114 | 99 | 137 | 171 | 183 | 115 | 131 | 182 | 103 | 102 | 179 | 135 | 104 |
| Bio 3 | 71 | 72 | 57 | 61 | 70 | 86 | 65 | 79 | 73 | 71 | 71 | 74 | 71 | 62 | 46 | 69 | 83 |
| Bio 4 | 2664 | 1112 | 2748 | 2595 | 1245 | 495 | 1920 | 1070 | 1632 | 1216 | 1370 | 1540 | 631 | 2060 | 7809 | 523 | 586 |
| Bio 5 | 145 | 236 | 314 | 309 | 155 | 316 | 174 | 232 | 191 | 156 | 167 | 200 | 329 | 347 | 410 | 354 | 337 |
| Bio 6 | -109 | 81 | 112 | 116 | -7 | 202 | -36 | 16 | -57 | -4 | -16 | -43 | 185 | 183 | 24 | 161 | 212 |
| Bio 7 | 254 | 155 | 202 | 193 | 162 | 114 | 210 | 216 | 248 | 160 | 183 | 243 | 144 | 164 | 386 | 248 | 260 |
| Bio 8 | 61 | 173 | 237 | 238 | 93 | 253 | 104 | 142 | 99 | 94 | 96 | 109 | 259 | 282 | 308 | 248 | 260 |
| Bio 9 | 16 | 148 | 186 | 186 | 64 | 262 | 58 | 119 | 64 | 66 | 64 | 70 | 258 | 264 | 247 | 251 | 272 |
| Bio 10 | 61 | 177 | 249 | 250 | 94 | 262 | 105 | 142 | 99 | 95 | 98 | 109 | 268 | 288 | 313 | 259 | 274 |
| Bio 11 | -7 | 148 | 179 | 185 | 64 | 250 | 58 | 116 | 58 | 66 | 64 | 70 | 252 | 237 | 114 | 247 | 260 |
| Bio 12 | 194 | 1668 | 1593 | 1566 | 964 | 2552 | 728 | 289 | 453 | 1134 | 743 | 430 | 2124 | 867 | 206 | 2415 | 3192 |
| Bio 13 | 79 | 276 | 194 | 191 | 210 | 260 | 161 | 75 | 107 | 249 | 158 | 106 | 310 | 177 | 33 | 385 | 459 |
| Bio 14 | 0 | 25 | 66 | 57 | 6 | 165 | 7 | 1 | 1 | 7 | 3 | 1 | 21 | 11 | 3 | 9 | 95 |
| Bio 15 | 159 | 62 | 29 | 33 | 84 | 13 | 81 | 107 | 105 | 83 | 89 | 107 | 61 | 79 | 48 | 69 | 46 |
| Bio 16 | 179 | 773 | 553 | 555 | 539 | 728 | 400 | 205 | 307 | 632 | 435 | 295 | 921 | 455 | 77 | 1083 | 1266 |
| Bio 17 | 1 | 101 | 246 | 213 | 27 | 573 | 28 | 7 | 9 | 34 | 16 | 8 | 100 | 36 | 13 | 49 | 363 |
| Bio 18 | 179 | 537 | 482 | 498 | 420 | 573 | 333 | 205 | 268 | 298 | 318 | 260 | 319 | 199 | 61 | 343 | 377 |
| Bio 19 | 1 | 101 | 322 | 266 | 27 | 646 | 28 | 9 | 9 | 34 | 16 | 8 | 353 | 62 | 62 | 1082 | 1233 |

UV irradiance 1: annual mean of ultraviolet irradiance , UV irradiance 2: mean of ultraviolet irradiance in the coldest semester, UV irradiance 3: mean of ultraviolet irradiance in the warmest semester; Bio = Bioclimatic variables, Bio 1: Annual Mean Temperature, Bio 2: Mean Diurnal Range, Bio 3: Isothermality, Bio 4: Temperature Seasonality, Bio 5: Maximum Temperature of Warmest Month, Bio 6: Minimum Temperature of Coldest Month, Bio 7: Temperature Annual Range, Bio 8: Mean Temperature of Wettest Quarter, Bio 9: Mean Temperature of Driest Quarter, Bio 10: Mean Temperature of Warmest Quarter, Bio 11: Mean Temperature of Coldest Quarter, Bio 12: Annual Precipitation, Bio 13: Precipitation of Wettest Month, Bio 14: Precipitation in the driest month, Bio 15: Precipitation Seasonality, Bio 16: Precipitation of Wettest Quarter, Bio 17: Precipitation of Driest Quarter, Bio 18: Precipitation of Warmest Quarter, Bio 19: Precipitation of Coldest Quarter. All temperature indexes are in °C × 10 and precipitation indexes are in mm. Irradiance is in W/m².

**Table B.** Allelic frequencies and Hardy-Weinberg Equilibrium results.

| Population | Country | *TP53* rs1042522 | | |  | *MDM2* rs2279744 | | |  | *MDM4* rs1563828 | | |  | *USP7* rs1529916 | | |  | *LIF*rs929271 | | |  | Reference |
| --- | --- | --- | --- | --- | --- | --- | --- | --- | --- | --- | --- | --- | --- | --- | --- | --- | --- | --- | --- | --- | --- | --- |
|  |  | C | G | n |  | T | G | n |  | A | G | n |  | G | A | n |  | T | G | n |  |  |
| Highlands(> 2.500 m.) |  |  |  |  |  |  |  |  |  |  |  |  |  |  |  |  |  |  |  |  |  |  |
| Amantani | Peru | 0.14 | 0.86 | 29 |  | **0.87** | **0.13** | 29 |  | 0.40 | 0.60 | 29 |  | 0.61 | 0.39 | 29 |  | 0.23 | 0.77 | 29 |  | This study |
| Anapia | Peru | 0.23 | 0.77 | 15 |  | **0.87** | **0.13** | 15 |  | 0.43 | 0.57 | 15 |  | 0.70 | 0.30 | 15 |  | 0.53 | 0.47 | 15 |  | This study |
| Cabanaconde | Peru | 0.12 | 0.88 | 17 |  | **0.88** | **0.12** | 17 |  | 0.26 | 0.74 | 17 |  | 0.91 | 0.09 | 17 |  | 0.68 | 0.32 | 17 |  | This study |
| Chivay | Peru | 0.17 | 0.83 | 18 |  | **0.86** | **0.14** | 18 |  | 0.33 | 0.67 | 18 |  | 1 | 0 | 18 |  | 0.64 | 0.36 | 18 |  | This study |
| Taquile | Peru | 0.08 | 0.92 | 43 |  | **0.95** | **0.05** | 43 |  | 0.37 | 0.63 | 43 |  | 0.77 | 0.23 | 43 |  | 0.56 | 0.44 | 43 |  | This study |
| Uros | Peru | 0.07 | 0.93 | 22 |  | **0.80** | **0.20** | 22 |  | 0.16 | 0.84 | 22 |  | 0.95 | 0.05 | 22 |  | 0.73 | 0.27 | 22 |  | This study |
| Yanke | Peru | 0.35 | 0.65 | 10 |  | 0.95 | 0.05 | 10 |  | 0.20 | 0.80 | 10 |  | 0.90 | 0.10 | 10 |  | 0.55 | 0.45 | 10 |  | This study |
| Aymara | Bolivia | 0.22 | 0.78 | 16 |  | 0.79 | 0.21 | 17 |  | 0.50 | 0.50 | 16 |  | 0.86 | 0.14 | 18 |  | 0.88 | 0.12 | 17 |  | This study |
| Quechua | Bolivia | **0.47** | **0.53** | 16 |  | 0.79 | 0.21 | 17 |  | 0.34 | 0.66 | 16 |  | 0.76 | 0.24 | 17 |  | **0.60** | **0.40** | 15 |  | This study |
| Lowlands (< 2.500 m.) |  |  |  |  |  |  |  |  |  |  |  |  |  |  |  |  |  |  |  |  |  |  |
| Andoas | Peru | 0.26 | 0.74 | 61 |  | **0.84** | **0.16** | 61 |  | 0.47 | 0.53 | 61 |  | 0.61 | 0.39 | 61 |  | 0.41 | 0.59 | 61 |  | This study |
| Guarani Kaiowa | Brazil | 0.06 | 0.94 | 16 |  | 0.93 | 0.07 | 16 |  | 0.34 | 0.66 | 16 |  | 0.66 | 0.34 | 16 |  | 0.44 | 0.56 | 16 |  | This study |
| Guarani Ñandeva | Brazil | 0.13 | 0.87 | 15 |  | 0.67 | 0.33 | 15 |  | 0.28 | 0.72 | 16 |  | 0.43 | 0.57 | 15 |  | 0.37 | 0.63 | 15 |  | This study |
| Karitiana | Brazil | 0.38 | 0.62 | 24 |  | 0.41 | 0.59 | 23 |  | ND | ND | ND |  | ND | ND | ND |  | ND | ND | ND |  | Sucheston *et al.* 2011 |
| Maya | Mexico | 0.17 | 0.83 | 23 |  | 0.36 | 0.64 | 21 |  | ND | ND | ND |  | ND | ND | ND |  | ND | ND | ND |  | Sucheston  *et al.* 2011 |
| Piapoco/Curripaco | Colombia | 0.08 | 0.92 | 12 |  | 0.19 | 0.81 | 13 |  | ND | ND | ND |  | ND | ND | ND |  | ND | ND | ND |  | Sucheston  *et al.* 2011 |
| Pima | USA | 0.35 | 0.65 | 24 |  | 0.31 | 0.69 | 21 |  | ND | ND | ND |  | ND | ND | ND |  | ND | ND | ND |  | Sucheston  *et al.* 2011 |
| Surui | Brazil | 0 | 1 | 17 |  | **0.68** | **0.32** | 20 |  | ND | ND | ND |  | ND | ND | ND |  | ND | ND | ND |  | Sucheston  *et al.* 2011 |

For the loci **in bold** deviations from the Hardy-Weinberg Equilibrium were detected.

ND: No data available.

**Table C. Binary logistic regression analyses results.**

|  | **B** | **SE** | **Wald** | **df** | **OR** | **CI 95 %** | ***­p*-value** |
| --- | --- | --- | --- | --- | --- | --- | --- |
| **Data set A** |  |  |  |  |  |  |  |
| *TP53* rs1042522 |  |  |  |  |  |  |  |
| GG (reference) | 0 |  |  |  | 1 |  |  |
| GC | -0.212 | 0.299 | 0.504 | 1 | 0.809 | 0.450-1.453 | 0.478 |
| CC | -0.233 | 0.500 | 0.217 | 1 | 0.792 | 0.297-2.111 | 0.641 |
| *MDM2* rs2279744 |  |  |  |  |  |  |  |
| TT (reference) | 0 |  |  |  | 1 |  |  |
| TG | -0.179 | 0.419 | 0.182 | 1 | 0.836 | 0.368-1.902 | 0.670 |
| GG | -0.361 | 0.432 | 0.698 | 1 | 0.697 | 0.299-1.626 | 0.403 |
| *MDM4* rs1563828 |  |  |  |  |  |  |  |
| GG (reference) | 0 |  |  |  | 1 |  |  |
| GA | -0.444 | 0.281 | 2.489 | 1 | 0.642 | 0.370-1.114 | 0.115 |
| AA | -0.552 | 0.375 | 2.172 | 1 | 0.576 | 0.276-1.200 | 0.141 |
| *USP7* rs1529916 |  |  |  |  |  |  |  |
| GG (reference) | 0 |  |  |  | 1 |  |  |
| GA | -0.875 | 0.285 | 9.415 | 1 | 0.417 | 0.238-0.729 | **0.002** |
| AA | -2.000 | 0.447 | 20.054 | 1 | 0.135 | 0.056-0.325 | **<0.001** |
| *LIF* rs929271 |  |  |  |  |  |  |  |
| TT (reference) | 0 |  |  |  | 1 |  |  |
| TG | -1.126 | 0.342 | 10.875 | 1 | 0.324 | 0.166-0.633 | **0.001** |
| GG | -1.310 | 0.368 | 12.681 | 1 | 0.270 | 0.131-0.555 | **<0.001** |
|  |  |  |  |  |  |  |  |
| **Data set B** |  |  |  |  |  |  |  |
| *TP53* rs1042522 |  |  |  |  |  |  |  |
| GG (reference) | 0 |  |  |  | 1 |  |  |
| GC | -0.427 | 0.238 | 3.220 | 1 | 0.653 | 0.409-1.040 | 0.073 |
| CC | -0.087 | 0.427 | 0.041 | 1 | 0.917 | 0.397-2.118 | 0.839 |
| *MDM2* rs2279744 |  |  |  |  |  |  |  |
| TT (reference) | 0 |  |  |  | 1 |  |  |
| TG | -1.523 | 0.305 | 24.868 | 1 | 0.218 | 0.120-0.397 | **<0.001** |
| GG | -1.745 | 0.322 | 29.407 | 1 | 0.175 | 0.093-0.328 | **<0.001** |

SE = standard error; df = degrees of freedom; OR = odds ratio; CI = confidence interval.

**Table D.** Locus interaction by the multifactor dimensionality reduction (MDR) approach.

| **Sample** | **Model** | **Testing accuracy** | ***p*-value*** | **Cross-validation consistency** |
| --- | --- | --- | --- | --- |
| **Data set A** |  |  |  |  |
|  | *USP7* | 0.6438 | 0.004 | 10/10 |
|  | *LIF*,*USP7* | 0.6409 | 0.004 | 10/10 |
|  | *LIF*,*TP53*,*USP7* | 0.6408 | 0.004 | 9/10 |
|  |  |  |  |  |
| **Data set B** |  |  |  |  |
|  | *MDM2* | 0.6688 | 0.001 | 10/10 |
|  | *TP53*, *MDM2* | 0.6690 | 0.001 | 10/10 |

*Evaluated using a 1000-fold permutation test to compare observed testing accuracies with those expected under the null hypothesis of null association.

**.** **h net.** Ne
